# A systematic review of direct oral anticoagulants for thromboprophylaxis in multiple myeloma

**DOI:** 10.1016/j.rpth.2025.102865

**Published:** 2025-04-22

**Authors:** Cătălina Codreanu, Tessa Elling, Nic J.G. M. Veeger, Wilfried W.H. Roeloffzen, Karina Meijer

**Affiliations:** 1 Department of Haematology, University of Groningen, University Medical Centre Groningen, Groningen, the Netherlands; 2 Department of Epidemiology, University of Groningen, University Medical Centre Groningen, Groningen, the Netherlands; 3 Department of Epidemiology, MCL Academy, Medical Centra Leeuwarden, Leeuwarden, the Netherlands

**Keywords:** anticoagulants, multiple myeloma, systematic review, thrombosis, venous thromboembolism

## Abstract

Multiple myeloma (MM) is associated with increased venous thromboembolism (VTE) risk. Current guidelines recommend aspirin or low-molecular-weight heparin for thromboprophylaxis depending on VTE risk. Nevertheless, VTE risks remain high: a recent meta-analysis reported an incidence of 6.2% during the entire MM course. Direct oral anticoagulants (DOACs) showed promising results in other malignancies. This systematic review provides an overview of evidence on DOAC thromboprophylaxis in MM. PubMed and Embase were searched up to November 21, 2023, for studies evaluating MM and DOAC thromboprophylaxis (PROSPERO: CRD42022376152). Two authors independently screened titles, abstracts, and texts, assessed bias using a modified version of the Newcastle Ottawa Scale and certainty of evidence with the Grading of Recommendations Assessment, Development and Evaluation approach, and performed data extraction and analysis. Seven articles comprising 416 patients with DOAC thromboprophylaxis were included, primarily involving newly diagnosed patients with MM (56.3%) receiving lenalidomide-based regimens (69.1%). Overall Newcastle Ottawa Scale study quality was moderate. Four studies reported follow-up duration ranging from 90 days after induction to 7 months. VTE proportions ranged from 0% to 23.5%, with 4 studies reporting 0%. The proportions of minor, clinically relevant nonmajor, and major bleeding ranged from 0% to 18.2%, 0% to 7.7%, and 0% to 4.5%, respectively. Arterial thrombosis proportions ranged from 0% to 2.9%. Only 2 studies reported on mortality (2% and 7.1%). Overall Grading of Recommendations Assessment, Development and Evaluation certainty of evidence was very low for all outcomes. Current evidence regarding routine DOACs in MM is insufficient, warranting further research to establish the DOAC thromboprophylaxis risk-to-benefit ratio in MM.

## Essentials

• Multiple myeloma has a high venous thromboembolism risk despite thromboprophylaxis.
• Evidence to support the use of direct oral anticoagulants in practice is insufficient.
• Venous thromboembolism proportions vary across studies, but heterogeneity and lack of follow-up data limit pooling.
• Well-designed studies are needed to establish the direct oral anticoagulant prophylaxis risk-to-benefit ratio in multiple myeloma.

## 1 Introduction

Multiple myeloma (MM) is associated with an increased risk of venous thromboembolism (VTE) and arterial thrombosis (AT), especially during the initial years following MM diagnosis [[1], [2], [3], [4], [5], [6]]. The thrombogenicity in patients with MM is caused by multiple factors, including disease-related aspects (eg, hyperviscosity and cytokine release), treatment-related factors (eg, placement of a central venous catheter, immunomodulatory drugs [IMiDs], high-dose steroids, and erythropoietin replacement therapy), as well as patient-related factors (eg, immobility, obesity, and a history of VTE) [3,[7], [8], [9], [10]].

Currently, 3 risk scores are available to evaluate VTE risk in patients with MM. The IMPEDE-VTE, SAVED, and PRISM scores take into account various patient, treatment, and disease characteristics [[11], [12], [13]]. The National Comprehensive Cancer Network, the International Myeloma Working Group (IMWG), and the European Myeloma Network guidelines recommend aspirin for low-risk and low-molecular-weight heparin (LMWH), vitamin K antagonist, or a direct oral anticoagulant (DOAC) for patients with high-risk MM [[14], [15], [16]]. The American Society of Hematology guidelines recommend aspirin or fixed low-dose vitamin K antagonist or LMWH for patients with MM with IMiD-based therapy [17]. However, the supporting evidence is limited, and a recent study showed a high VTE risk for patients with MM receiving IMiD-based therapy despite implementation of the IMWG guidelines [18]. The incidence of VTE remains high, affecting approximately 6.2% of patients during the entire disease course according to a recent meta-analysis [19]. This emphasizes the ongoing unmet clinical need to determine the most optimal strategy for preventing thrombosis in patients with MM.

DOACs offer a promising alternative to current antithrombotic approaches. Their oral administration provides a more patient-friendly therapy compared with the subcutaneous administration of LMWH, potentially enhancing treatment compliance. Furthermore, DOACs have shown a significant benefit in preventing VTE in high-risk ambulatory cancer patients, with a low incidence of major bleeding (MB). Nevertheless, the use of placebos in these randomized controlled trials (RCTs) resulted into an underrepresentation of patients with MM, who inherently required thromboprophylaxis [[20], [21], [22]]. Additionally, observational studies have shown promising results regarding the efficacy and safety of DOAC therapy in patients with MM [[23], [24], [25], [26], [27], [28], [29]].

A recent systematic review and meta-analysis by Costa et al. [30] focused on the comparison of VTE and bleeding risk in patients with MM receiving either DOAC or aspirin. They reported a lower odd of VTE in patients receiving DOAC thromboprophylaxis with similar bleeding odds. Although an important comparison, it may provide an incomplete representation of the MM population, as it might exclude high-risk patients who receive LMWH as thromboprophylaxis [30]. Therefore, an overview of all patients with MM irrespective of their VTE risk is needed. This systematic review aimed to provide an overview of the available evidence regarding the incidence of VTE and bleeding in patients with MM receiving DOAC thromboprophylaxis irrespective of their VTE risk.

## 2 Methods

This systematic review was registered in the International Prospective Register of Systematic Reviews (PROSPERO) (CRD42022376152), reported according to the Preferred Items for Systematic Reviews and Meta-Analyses guidelines [31].

### 2.1 Study inclusion criteria

We included studies reporting on the incidence of thrombotic events, bleeding, and all-cause mortality in adult patients with MM receiving DOACs as thromboprophylaxis. Patients during any MM treatment line were eligible for inclusion (ie, newly diagnosed or relapse of disease). The outcomes of interest were VTE, divided per deep vein thrombosis (DVT), pulmonary embolism (PE), and any other VTE as reported by individual studies: bleeding events, divided per MiB, clinically relevant nonmajor bleeding (CRNMB), and MB; arterial thromboembolism (AT), categorized as ischemic stroke, myocardial infarctions, and any other AT as reported by individual studies; and all-cause mortality, expressed as number of deaths. If bleeding events were classified according to the International Society on Thrombosis and Haemostasis (ISTH) criteria, we accepted this classification [32]. If bleeding events were not classified according to these criteria, but the description of the event was provided, the authors decided on a case-by-case basis to which ISTH category to attribute the event.

Only peer-reviewed cohort studies and RCTs were considered for inclusion. In RCTs and cohort studies, only the group receiving a DOAC was included in the analysis. Cross-sectional studies and case-reports were excluded. Moreover, studies with a cohort of 10 patients or less were excluded as we assumed that these would not be of sufficient quality.

### 2.2 Search strategy and used databases

In consultation with a medical librarian of the University of Groningen, a comprehensive search syntax was designed. MeSH terms and synonyms for “multiple myeloma” and “direct oral anticoagulant” were used. The search syntax is available in the Supplementary Material. Consequently, a systematic search was conducted by 2 reviewers (C.C. and T.E.) in PubMed and Embase on November 1, 2022. A filter for the English language was used. Gray literature was not considered for inclusion. There was no restriction on publication year. Data management, deduplication, and article selection were performed in Rayyan [33]. Additionally, a manual search of the reference lists of retrieved articles and relevant systematic reviews and meta-analyses was performed. At the completion of the study (November 21, 2023) an update search was performed.

### 2.3 Article selection

The article screening and selection process was independently performed by 2 reviewers (C.C. and T.E.) in 2 stages. Articles were included for full-text screening if 1 or both reviewers screened the title/abstract positively. During full-text screening, discrepancies were resolved through discussion with a third reviewer (K.M.).

### 2.4 Data collection

Data were independently extracted by 2 reviewers (C.C. and T.E.) in a predesigned format in Excel. Subsequently, discrepancies were checked and resolved in consultation with a third reviewer (K.M.). The following data were extracted: study design, cohort demographics (ie, age and sex/gender as reported by the included articles), patient comorbidities (ie, cardiovascular diseases, chronic kidney failure, diabetes mellitus, and Crohn’s disease), and risk factors for thromboembolic events (ie, a history of VTE and/or AT; thrombophilia; tunneled line or central catheter *in situ*; surgery in the past 4 weeks, hyperviscosity, use of oral contraceptives; smoking status; hypercholesterolemia). Furthermore, data about MM therapy (ie, type of therapy), MM stage (ie, [revised] international staging system), type of DOAC thromboprophylaxis, type and number of VTE events, type and number of AT events, type and number of bleeding events, number of deaths, and follow-up length. In case of missing data, the respective authors were contacted for further information and if provided, it was included in the study.

### 2.5 Risk of bias assessment

Two reviewers (C.C. and T.E.) independently assessed the risk of bias of the included studies. Subsequently, discrepancies were checked and resolved in consultation with a third reviewer (K.M.). A modified version of the Newcastle Ottawa Scale (NOS) was used (Supplementary Material) [34]. The following items were assessed to provide a final score of 0 to 6: representativeness of the cohort, ascertainment of exposure, demonstration that the outcome of interest was not present at study initiation, assessment of the outcome, duration of follow-up, and adequacy of follow-up. The items selection of the nonexposed cohort and comparability were omitted, as we did not compare thromboprophylaxis regimens. A higher score signified a higher level of study quality.

### 2.6 Data analysis

Data analysis was performed in R Studio v4.1.3 by R Core Team. All analyses were done independently in duo by C.C. and T.E. and subsequently checked. Study characteristics and outcome variables were reported as number and percentages, mean and SD, median and IQR, or minimum and maximum. In case only median values were provided, they were converted to mean [35].

Two people (C.C. and T.E.) independently assessed the certainty of evidence. Discrepancies were checked and resolved in consultation with a third reviewer (K.M.). We assessed the certainty of evidence at the outcome level using the Grading of Recommendations Assessment, Development and Evaluation approach on 5 domains: risk of bias, imprecision, inconsistency, indirectness, and publication bias [36]. We assessed the certainty of evidence as high, moderate, low, or very low.

## 3 Results

The search in PubMed and Embase yielded 524 and 1420 results, respectively (Figure). After deduplication, 1468 articles were screened by title and abstract. Subsequently, 115 articles were selected for full-text screening. Finally, 7 articles comprising 8 cohorts with 416 patients were included in the final review [[23], [24], [25], [26], [27], [28], [29]]. A manual search of the reference lists of retrieved articles and relevant systematic reviews and meta-analyses did not reveal additional studies. Most studies were excluded because they did not use DOAC thromboprophylaxis or did not report the results separately for DOACs. No RCTs were identified that met the inclusion criteria.

**Figure:**
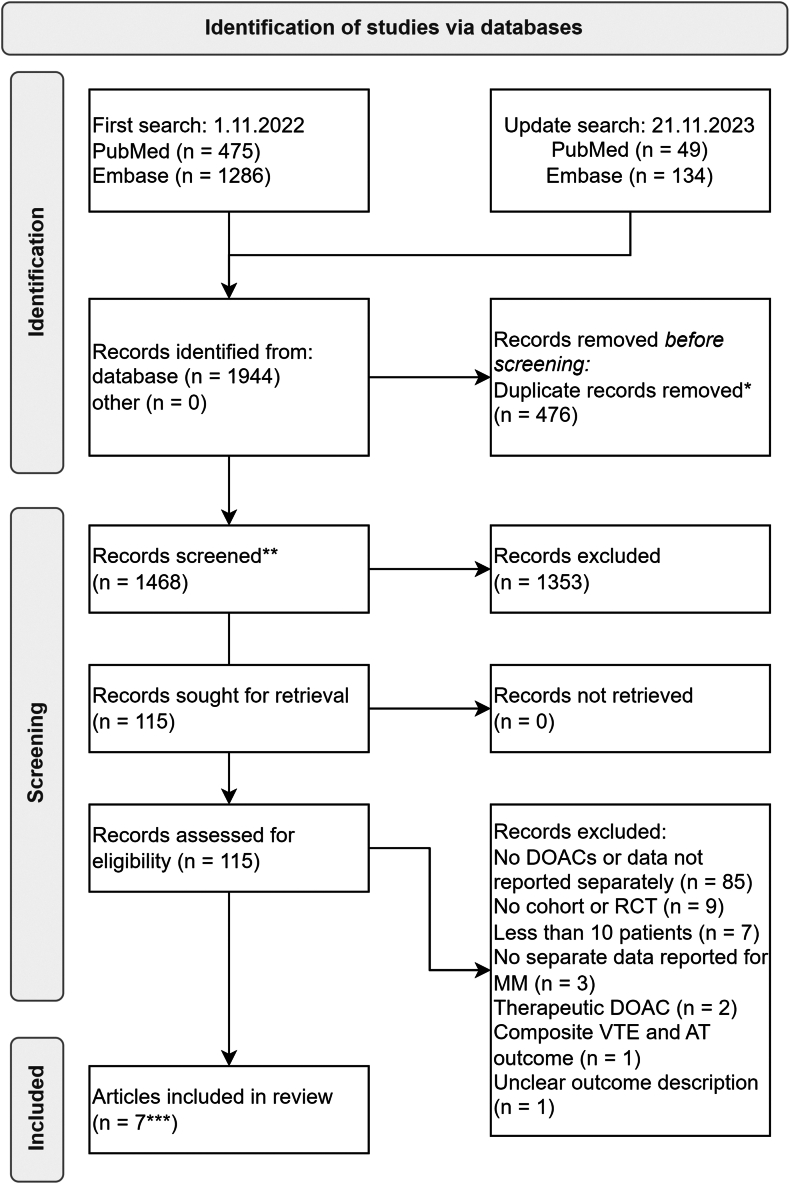
Flowchart of article selection and inclusion. ∗443 duplicates removed from first search and 33 duplicates from second search. ∗∗1318 articles screened during first search and 150 articles during second search. ∗∗∗6 articles included from first search and 1 article from the update search. AT, arterial thromboembolism; DOAC, direct oral anticoagulant; MM, multiple myeloma; RCT, randomized controlled trial; VTE, venous thromboembolism. Records identified from other: from manual screening of appropriate systematic review references.

### 3.1 Quality assessment

Overall study quality scored with the modified NOS was moderate, with a median of 3 (IQR, 2.0-4.3). Most studies did not report on the follow-up length and completeness (4/8 cohorts [50%]) or on whether the outcome was not present at inclusion (3/8 cohorts [37.5%]) (Supplementary Table S1).

### 3.2 Study characteristics

An overview of study characteristics is provided in Tables 1 and 2. Few studies had a complete description of the patient population, and very little data were available on comorbidities. Four articles (5 cohorts) reported on apixaban [[23], [24], [25],^28^], 2 reported on rivaroxaban [26,29], and 1 did not specify what type of DOAC was used [27]. There were 5 retrospective [23,[26], [27], [28], [29]] and 3 prospective [24,25,28] cohorts (1 study [28] reported on a prospective and retrospective cohort). Three studies [[23], [24], [25]] reported a numerical follow-up length, which ranged from 6 to 7 months. Two of these studies reported on completeness of follow-up, with 12% of patients in each study stopping with apixaban thromboprophylaxis early [24,25]. Two studies reported on the duration of DOAC thromboprophylaxis, namely 168 days [24] and 3.8 months [23]. Three studies did not report follow-up durations [[27], [28], [29]] or provided nonnumerical follow-up information, such as from the initiation of therapy until 90 days after completion of induction therapy [26]. Five studies reported bleeding events according to the ISTH criteria [[23], [24], [25], [26],^28^]. One study reported 0 bleeding events [29]. One study did not prespecify the use of the ISTH criteria, however, descriptively reported only 2 MiB events [27]. Four studies specified how they had diagnosed thrombosis [[24], [25], [26],^29^].

**Table 1:** Patient characteristics.

Characteristic		No. cohorts	No. patients
No. of patients	416	8	416
Age (y), range of means	62.8-69.8	3/8	224
Female patients, *n* (%)	147 (48.0)	4/8	306
History of VTE, *n* (%)	17 (7.6)	3/8	224
MM stage, *n* (%)		5/8	323
Newly diagnosed	182 (56.3)		
Consolidation	10 (3.1)		
Relapsed/refractory	111 (34.4)		
Maintenance	20 (6.2)		
MM therapy, *n* (%)		5/8	317
Lenalidomide based	219 (69.1)		
Bortezomib/lenalidomide/dexamethasone	4 (1.3)		
CCRD	8 (2.5)		
Lenalidomide/cyclophosphamide/dexamethasone	4 (1.3)		
Daratumumab/lenalidomide/dexamethasone	1 (0.3)		
Elotuxumab/lenalidomide/dexamethasone	3 (0.9)		
Ixazomib/lenalidomide/dexamethasone	3 (0.9)		
Carfilzomib/lenalidomide/dexamethasone	86 (27.1)		
Lenalidomide/dexamethasone	102 (32.2)		
Lenalidomide monotherapy	8 (2.5)		
Thalidomide based	46 (14.5)		
CTD/CTDa	19 (6.0)		
Bortezomib/thalidomide/dexamethasone	27 (8.5)		
Pomalidomide based	21 (6.6)		
Bortezomib/pomalidomide/dexamethasone	1 (0.3)		
Daratumumab/pomalidomide/dexamethasone	5 (1.6)		
Elotuzumab/pomalidomide/dexamethasone	1 (0.3)		
Cyclophosph/pomalidomide/dexamethasone	1 (0.3)		
Ixazomib/pomalidomide/dexamethasone	2 (0.6)		
Pomalidomide monotherapy	2 (0.6)		
Pomalidomide/dexamethasone	4 (1.3)		
Carfilzomib/pomalidomide/dexamethasone	5 (1.6)		
*No-IMiDs*	20 (6.3)		
Melphalan/prednisolone/dexamethasone	20 (6.3)		
*IMiDs (not specified)*^a^	11 (3.5)		
DOAC thromboprophylaxis, *n* (%)		8/8	416
Apixaban	306 (73.6)		
Rivaroxaban	99 (23.8)		
Not specified	11 (2.6)		

Patient comorbidities, thrombosis risk factors, and MM stage were not consequently reported and thus not reported.

CCRD, carfilzomib, cyclophosphamide, lenalidomide, dexamethasone; CTD(a), cyclophosphamide, thalidomide, dexamethasone (attenuated); DOAC, direct oral anticoagulant; IMiD, immunomodulatory drug; MM, multiple myeloma; VTE, venous thromboembolism.

a This article did not report per regimen.

**Table 2:** Study characteristics.

Reference	Year	Study design	Sample size	Age^a^	Female (%)	History of VTE (%)	MM therapy (%)	MM stage (%)	Type of DOAC (dose)	Designed length of follow-up.
Sayar et al.^b^ [28]	2022	RS	22	NR	NR	NR	NR^c^	NR	Apixaban (2.5 mg 2 dd)	NR^d^
Sayar et al.^b^ [28]	2022	PS	60	NR	NR	NR	NR^c^	NR	Apixaban (2.5 mg 2 dd)	NR^d^
Parnes et al. [27]	2022	RS	11	NR	NR	NR	NR	NR	NR	NR^d^
Piedra et al. [26]	2022	RS	82	<65-54 (66%) ≥65-28 (34%)	54.9	NR	Firstline: 100	ISS I: 54.9 ISS II: 32.9 ISS III: 12.2	Rivaroxaban (10 mg 1 dd)	From the first day of therapy until 90 d after completion of induction therapy.
Cornell et al. [25]	2020	PS	50	63	50.0	0	Firstline: 4.0 Consolidation: 20.0 Maintenance: 40.0 Relapse: 36.0	R-ISS I: 46 R-ISS II: 34.6 R-ISS III: 16	Apixaban (2.5 mg 2 dd)	6 mo
Pegourie et al. [24]	2019	PS	104	70	50.0	14.4	Firstline: 10.6 Relapse: 89.4	NR	Apixaban (2.5 mg 2 dd)	Received apixaban for 6 mo and were monitored for 7 mo
Storrar et al. [23]	2019	RS	70	66	35.7	2.9	Firstline: 100	NR	Apixaban (2.5 mg 2 dd)	The first 4 mo of therapy or until treatment was completed—whichever was shorter, and for a maximum of 6 mo
Li et al. [29]	2023	RS	17	NR	NR	NR	Firstline: 100	NR	Rivaroxaban (NR)	NR^d^

dd, dose daily; DOAC, direct oral anticoagulant; ISS, international staging system for multiple myeloma; MM, multiple myeloma; NR, not reported; PS, prospective cohort; RS, retrospective cohort; R-ISS, revised international staging system; VTE, venous thromboembolism.

a Presented as mean or median.

b Sayar et al [28] consisted of 2 cohorts, retrospective and prospective cohorts.

c All patients were patients classified in the high-thrombotic risk group. No further information was given.

d Follow-up was reported only for the entire cohort and not for the DOAC subgroup.

From the studies with available data on the MM line of treatment, 56.3% of patients received firstline therapy [[23], [24], [25], [26],^29^]. Five of 8 cohorts reported on the therapy that patients with MM with DOAC thromboprophylaxis received [[23], [24], [25], [26], [27], [28], [29]]. The majority of patients received lenalidomide/dexamethasone (32.2%) and carfilzomib/lenalidomide/dexamethasone (27.1%) (Table 1). Almost 7% received a pomalidomide-based regimen, 15% received a thalidomide-containing regimen, and 69% received lenalidomide-based regimens. Only 3 of 7 articles reported on history of VTE, 7.6% of patients had a VTE in their medical history (Table 2).

Despite the expected heterogeneity among included studies, no meta-regression analysis could be done because of the lack of information regarding patient and study characteristics. Moreover, because of the lack of follow-up data, the event risks could not be pooled in a reliable way.

### 3.3 Reason to prescribe DOACs

The choice to prescribe DOACs as prophylaxis differed among the included studies. In 4 of the included studies, apixaban was prescribed in patients with high VTE risk and/or patients receiving IMiD-based therapy [24,25,27,28]. In 2 retrospective studies DOAC thromboprophylaxis represented standard-of-care [23,26]. In 1 retrospective study the reason for different thromboprophylaxis was not elaborated upon [29].

### 3.4 Methods for VTE risk stratification

Two studies did not report VTE risk stratification for the patients with MM receiving DOACs [23,29]. One study defined high VTE risk based on a list of VTE risk factors [28]. Two studies reported VTE risk based on the IMWG criteria [24,27]. Two studies used VTE risk scores, namely the SAVED score [26] and the IMPEDE-VTE score [25]. In total, 169 patients were categorized as low VTE risk, 106 as high risk, and 32 as intermediate risk, and for 109 patients, the VTE risk was not defined.

### 3.5 VTE estimates

All studies reported on VTE proportion, with estimates ranging from 0% to 23.5% (Table 3). In total, 10 events, ie, 9 DVTs (1 internal jugular vein thrombosis, 3 distal DVTs, 2 proximal DVTs, and 3 not specified) and 1 PE, occurred in 416 patients [[23], [24], [25], [26], [27], [28], [29]]. Four of 7 articles reported 0 events [23,25,27,28]. For 3 studies that reported follow-up, the absolute VTE risks ranged from 0% per 6-month follow-up to 1.9% per 7-month follow-up (Table 3) [[23], [24], [25]].

**Table 3:** Outcome estimates per included study.

Study	Sample size (*n*)	VTE, *n* (%)	MB, *n* (%)	CRNMB, *n* (%)	MiB, *n* (%)	AT, *n* (%)	Death, *n* (%)	Follow-up (d)
Sayar et al. (RS) [28]	22	0	1 (4.5)	0	1 (4.5)	NA	NA	NA
Sayar et al. (PS) [28]	60	0	0	1 (1.7)	5 (8.3)	NA	NA	NA
Parnes et al. [27]	11	0	0	0	2 (18.2)	NA	NA	NA
Piedra et al. [26]	82	4 (4.9)	0	NA	1 (1.2)	NA	NA	NA^a^
Cornell et al. [25]	50	0	0	3 (6.0)	NA	0	1 (2.0)	180
Pegourie et al. [24]	104	2 (1.9)	1 (1.0)	8 (7.7)	NA	0	NA	210
Storrar et al. [23]	70	0	1 (1.4)	0	NA	2 (2.9)	5 (7.1)	180
Li et al. [29]	17	4 (23.5)	0	0	0	0	NA	NA

AT, arterial thrombosis; CRNMB, clinically relevant nonmajor bleeding; d, days; MB, major bleeding; MiB, minor bleeding; NA, not available; PS, prospective; RS, retrospective; VTE, venous thromboembolism.

a No numeric value for follow-up available; please see Table 2.

Five studies reported on patients with MM with firstline therapy. In these studies, the VTE proportion ranged from 0% to 23.5%. In total, 8 events occurred in 182 patients [[23], [24], [25], [26],^29^]. Pegourie et al. [24] and Cornell et al. [25] reported VTE incidences of 2.2% during 7 months’ follow-up and 0% during 6 months’ follow-up for patients being treated for a MM relapse, respectively. In total, 2 events occurred in 111 patients [24,25]. Cornell et al. [25] also reported on 10 patients receiving consolidation therapy and 20 patients receiving maintenance therapy. No VTE events were reported for this cohort [25].

### 3.6 Bleeding estimates

All studies reported on MB (Table 3), with proportions ranging from 0% to 4.5%. Five of 8 cohorts experienced zero events [[25], [26], [27], [28], [29]]. In total, 3 MB events (1 traumatic subdural hematoma, 1 severe epistaxis requiring blood transfusion, and 1 melena) occurred in 416 patients [[23], [24], [25], [26], [27], [28], [29]]. For the 3 studies that reported follow-up, the absolute MB risk was 0% in 6 months [25], 1.0% in 7 months [24], and 1.4% in 6 months [23] (Table 3).

Six studies reported on CRNMB proportions [[23], [24], [25],[27], [28], [29]], ranging from 0% to 7.7%. In total, 12 patients experienced 14 events (Table 3). Events included an episode of hemoptysis secondary to COVID-19 [28], epistaxis, and 2 ecchymoses of the extremities [25], 2 epistaxis, 4 gastrointestinal bleedings in 2 patients, 3 genitourinary bleedings, and 1 hematoma [24]. For the studies that reported follow-up, the absolute CRNMB risk was 0% in 6 months [23], 7.7% in 7 months [24], and 6.0% in 6 months [25] (Table 3).

Four studies reported on MiB proportions (Table 3), ranging from 0% to 18.2% [[26], [27], [28], [29]]. In total, 9 of 112 patients experienced an event (1 epistaxis, 1 grade 1 gastrointestinal bleed, and 7 not specified).

### 3.7 AT estimates

Four studies reported on AT proportions (Table 3) [[23], [24], [25],^29^], ranging from 0% to 2.9%. Two patients experienced an event (1 stroke and 1 myocardial infarction) of 241 patients. For 3 studies that reported follow-up, absolute AT risks were 0% in 7 months [24], 0% in 6 months [25], and 2.9% in 6 months [23] (Table 3).

### 3.8 Mortality

Two studies reported on mortality (Table 3), namely 2% and 7.1% within a 6-month follow-up. Across these 2 studies with comparable follow-up, 6 of 120 patients died [23,25].

### 3.9 Certainty of evidence

Overall, certainty of evidence was very low for all outcomes. Publication bias could not be assessed with a funnel plot because of the small number of included studies. Supplementary Table S2 provides a summary of the certainty of evidence per outcome with explanation.

## 4 Discussion

### 4.1 Principal findings

This systematic review identified 7 articles that evaluated the efficacy and safety of DOACs as thromboprophylaxis in patients treated for MM. Most of the included cohorts assessed newly diagnosed patients with MM with a lenalidomide-based regimen. In these cohorts, 2.4% experienced a VTE and 0.7% experienced MBs. In 4 studies that provided either descriptive or numerical follow-up data, with follow-up periods ranging from 90 days after induction therapy to 7 months, the risk of VTE varied between 0% and 4.9%, while the risk of MB ranged from 0% to 1.4% [[23], [24], [25], [26]]. Among the studies that reported on CRNMB and MiB proportions, 3.6% of the patients experienced CRNMBs and 4.7% experienced MiBs. In the 4 studies that reported on AT proportions, 0.8% experienced an event. Only 2 studies had data available on mortality, with 5% experiencing a fatal event within a follow-up period of 6 months.

### 4.2 Comparison with previous studies

Previous studies have evaluated the occurrence of VTE, bleeding events, AT, and mortality in patients with MM receiving thromboprophylaxis with LMWH, warfarin, or aspirin. Follow-up varied from 6 to 60 months [18,19,[37], [38], [39]]. In these studies, the type of thromboprophylaxis was at the discretion of the investigator or in accordance with the IMWG guidelines [15].

The studies included in this systematic review observed VTE proportions ranging from 0% to 23.5% [[23], [24], [25], [26], [27], [28], [29]]. Most studies observed no VTE events [23,25,27,28]. Nevertheless, it is difficult to interpret these results in the absence of detailed information on follow-up duration. Previous RCTs reported DVT risks ranging from 1.1% to 3.6% for aspirin, 1.2% to 2.7% for LMWH, and 6.4% for warfarin and PE risks ranging from 1.7% to 1.8% for aspirin, 0% for LMWH, and 1.8% for warfarin, for newly diagnosed patients with MM on an IMiD-based regimen within 6 months of starting induction therapy [37,38]. A meta-analysis by Carrier et al. [39], including RCTs and prospective cohort studies, reported VTE rates ranging from 0.5 to 2.6 per 100 patient-cycles for newly diagnosed patients with MM receiving thalidomide and any thromboprophylaxis, and VTE incidence rates of 0.7 per 100 patient-cycles for lenalidomide and any prophylaxis [39].

In previous RCTs involving patients with newly diagnosed MM receiving IMiD-based therapy followed up for 6 months after start of induction therapy, MBs risks ranged from 0% to 1.4% for aspirin. No MB events were reported in patients receiving LMWH or warfarin. Additionally, risk estimates for MiB events were 2.7%, 1.4%, and 0.5% for aspirin, LMWH, and warfarin, respectively [37,38]. Larocca et al. [38] reported a MiB risk of 0.6% for patients receiving LMWH as thromboprophylaxis. In this systematic review, similar MB proportions were reported among the included studies, ranging from 0% to 4.5%, with 5 cohorts reporting 0 MBs. The reported MiB proportions, ranging from 0% to 18.2%, among the included studies appear to be slightly higher than those reported in previous literature for current thromboprophylaxis [[26], [27], [28], [29],^37^,^38^].

For standard-of-care thromboprophylaxis, a meta-analysis reported a 1-year cumulative incidence of 2.7% for AT. This meta-analysis included patients with MM with both newly diagnosed disease and recurrence of disease [5]. In our systematic review, AT proportions ranged from 0% to 2.9%, with 3 of 4 studies reporting 0% [[23], [24], [25],^29^].

For current thromboprophylaxis, 2 RCTs reported cases of mortality. There were no reported deaths during the follow-up period in the RCT conducted by Larocca et al. [38]. Palumbo et al. [37] documented 4 deceased patients (0.6%). In our systematic review, 2 studies reported on mortality (2.0% and 7.1%) [23,25]. MM is a chronic disease and the 5-year relative survival of patients with MM under the age of 80 reaches as high as 62.4% due to improving treatment strategies. This means that more and more patients succumb to comorbidities or late therapy toxicity and not to the disease itself [40].

DOAC thromboprophylaxis has also been studied in high-risk ambulatory cancer patients. However, in these RCTs, the use of placebos resulted in an underrepresentation of patients with MM, who inherently require thromboprophylaxis. The VTE risk appears to be lower than in patients with MM, ranging from 1.0% to 3.5% within a treatment period of 180 days. This difference may be attributed to the relatively lower baseline risk in non-MM cancer patients. Additionally an increased bleeding risk of 2% in high-risk ambulatory cancer patients is observed, which might be explained by individual risk factors for bleeding events, such as hypertension or fall risk [20,22].

The 2023 systematic review and meta-analysis by Costa et al. [30] reported lower VTE odds in patients with MM with DOAC thromboprophylaxis compared to aspirin (odds ratio, 0.33; 95% CI, 0.16-0.68) [30]. To our knowledge, studies comparing DOACs with LMWH are however unfortunately unavailable. Although current evidence suggests a preference for DOACs instead of aspirin for patients with high-risk MM, no conclusions can be made regarding LMWH vs DOACs.

It is challenging to compare our systematic review with previous studies due to the high degree of heterogeneity among patients with MM. Patient-specific and treatment-specific risk factors increase the risk of VTE. Furthermore, newly diagnosed patients with MM exhibit a higher VTE risk compared with previously treated patients with MM. Risk stratification with the SAVED, IMPEDE-VTE, or PRISM scores is based on these risk factors [[10], [11], [12], [13],^41^]. It is unclear whether this was systematically evaluated in the included cohorts. The patients were likely high risk, as they received anticoagulants instead of aspirin. Therefore, DOACs may be the best option for high-risk patients, but a clear patient profile to guide clinician’s decisions is lacking.

DOACs could be associated with a higher patient satisfaction compared with LMWH, which must be administered subcutaneously daily. This may be considered burdensome for patients and may reduce therapy adherence. Previous studies looking at patients requiring thromboprophylaxis because of cancer-associated thrombosis showed a strong preference for oral administration [42,43]. Patients justified their choice based on easiness of use and less pain experienced [42]. Patient satisfaction and use of preferred treatment method results in better adherence and overall reduction in recurrence of thrombosis [44].

### 4.3 Strengths and limitations

Our systematic review was comprehensive and systematic, with a thorough search of multiple electronic databases and hand searching of the reference list of the included articles. We provided a comprehensive overview of all available evidence, and we described in detail the included studies. We critically assessed the quality of included studies with the NOS risk of bias assessment and the certainty of evidence for all outcomes with the Grading of Recommendations Assessment, Development and Evaluation tool. This helps in translating the study results to clinical practice.

Several limitations of our study warrant consideration. Our ability to assess the absolute risks of thrombosis and bleeding is limited by the lack of follow-up data in current literature, making it challenging to evaluate the relevance of reported outcomes without considering the time context. Additionally, substantial differences were observed among the included studies regarding VTE risk stratification, reason for DOAC prescription and patient characteristics, which made further generalizability of the results for clinical practice and comparison with previous literature difficult. The nonrandomized nature of the studies could introduce potential bias. Moreover, patients with a higher VTE risk could be more likely to be prescribed an anticoagulant and as such this could introduce selection bias in this analysis. Finally, all described outcomes were rated as having a very low quality of evidence.

### 4.4 Clinical relevance and implications

The high incidence of VTE and AT in patients with MM despite current anticoagulation regimens, especially during firstline therapy for newly diagnosed MM, emphasizes the need for improved thromboprophylaxis. DOACs might be a promising alternative for LMWH and aspirin to reduce the VTE risk in patients with MM at the possible cost of slightly higher bleeding risks. Furthermore, the oral administration of DOACs provides a more patient-friendly therapy compared with the subcutaneous administration of LMWH. However, further evidence is essential. Large, high-quality RCTs or registry studies are needed to evaluate the efficacy and safety of DOACs in patients with MM.

## 5 Conclusion

In conclusion, DOACs may represent a promising alternative to current thromboprophylaxis for patients with high-risk MM. However, high-quality robust evidence regarding the use of routine DOAC thromboprophylaxis specifically in patients with MM is lacking. Further research is needed to establish the risk-to-benefit ratio of DOAC prophylaxis in patients with MM.
