# Supplementary material for: A systematic review of direct oral anticoagulants for thromboprophylaxis in multiple myeloma
Source: Res Pract Thromb Haemost. 2025 Apr 22;9(3):102865. doi: 10.1016/j.rpth.2025.102865 (PMC12140949; doi:10.1016/j.rpth.2025.102865)
Supplement: Supplementary Material [file mmc1.docx]

**Supplementary material**

1. **Search strategy**

| **PubMed** |
| --- |
| ("Multiple Myeloma"[Mesh] OR myeloma*[tiab] OR “plasma cell dyscrasia*”[tiab] OR "plasma cell proliferative disease*"[tiab] OR "plasma cell leukemia*"[tiab] OR “monoclonal gammopath*”[tiab])  AND  ("Factor Xa Inhibitors"[Mesh] OR "Factor Xa Inhibitors" [Pharmacological Action] OR "Antithrombins" [Pharmacological Action] OR “anti coagul*”[tiab] OR anticoagul*[tiab] OR “factor xa inhibitor*”[tiab] OR “direct thrombin inhibitor*”[tiab] OR doac*[tiab] OR noac*[tiab] OR apixaban[tiab] OR rivaroxaban[tiab] OR dabigatran[tiab] OR edoxaban[tiab] OR betrixaban[tiab] OR antithrombo*[tiab] OR “anti thrombo*”[tiab] OR (thrombo*[tiab] AND (prophyla*[tiab] OR prevent*[tiab])) OR thromboprophyla*[tiab]) |
| **EMBASE** |
| (‘myeloma’/exp OR ‘myeloma*’:ab,ti OR ‘plasma cell dyscrasia*’:ab,ti OR ‘plasma cell proliferative disease*’:ab,ti OR ‘plasma cell leukemia*’:ab,ti OR ‘monoclonal gammopath*’:ab,ti)  AND  (‘blood clotting factor 10a inhibitor’/exp OR ‘antithrombin’/exp OR ‘anti coagul*’:ab,ti OR ‘anticoagul*’:ab,ti OR ‘factor xa inhibitor*’:ab,ti OR ‘direct thrombin inhibitor*’:ab,ti OR ‘doac*’:ab,ti OR ‘noac*’:ab,ti OR ‘apixaban’:ab,ti OR ‘rivaroxaban’:ab,ti OR ‘dabigatran’:ab,ti OR ‘edoxaban’:ab,ti OR ‘betrixaban’:ab,ti OR ‘antithrombo*’:ab,ti OR ‘anti thrombo*’:ab,ti OR (‘thrombo*’:ab,ti AND (‘prophyla*’:ab,ti OR ‘prevent*’:ab,ti)) OR ‘thromboprophyla*’:ab,ti)  NOT  ('conference abstract'/it OR ‘erratum’/it OR ‘note’/it)  AND  [English]/lim |

1. **Modified Newcastle Ottawa Scale**

COHORT STUDIES

Note: A study can be awarded a maximum of one star for each numbered item within the Selection and Outcome categories. A maximum of two stars can be given for Comparability

**Selection**

1) Representativeness of the exposed cohort

1. truly representative *
2. somewhat representative *
3. selected group
4. no description of the derivation of the cohort

~~2) Selection of the non-exposed cohort~~

1. ~~drawn from the same community as the exposed cohort *~~
2. ~~drawn from a different source~~
3. ~~no description of the derivation of the non exposed cohort~~

3) Ascertainment of exposure

1. secure record (eg surgical records) *
2. structured interview *
3. written self report
4. no description
5. other

4) Demonstration that outcome of interest was not present at start of study

1. yes *
2. no

**~~Comparability~~**

~~1) Comparability of cohorts on the basis of the design or analysis~~

1. ~~study controls for age, sex and marital status *~~
2. ~~study controls for other factors (list) ________________________*~~
3. ~~cohorts are not comparable on the basis of the design or analysis controlled for confounders~~

**Outcome**

1) Assessment of outcome

1. independent blind assessment *
2. record linkage *
3. self report
4. no description
5. other

2) Was follow-up long enough for outcomes to occur

1. yes *
2. no

3) Adequacy of follow up of cohorts

1. complete follow up - all subjects accounted for *
2. subjects lost to follow up unlikely to introduce bias *
3. follow up rate less than 80% and no description of those lost
4. no statement
5. **Quality assessment**

**Table S1**. Risk of Bias assessment

| 1^st^ author | Year | Selection | | | Outcome | | | Total score |
| --- | --- | --- | --- | --- | --- | --- | --- | --- |
|  |  | Representativeness of cohort | Ascertainment of exposure | Demonstration that outcome was not present at start of study | Assessment of outcome | Was follow-up long enough for outcomes to occur | Adequacy of follow-up |  |
| Sayar^1^ | 2022 | * | * | 0 | 0 | 0 | 0 | 2/6 |
| Sayar^2^ | 2022 | * | * | 0 | 0 | 0 | 0 | 2/6 |
| Parnes | 2022 | * | * | 0 | 0 | 0 | 0 | 2/6 |
| Piedra | 2022 | * | * | 0 | * | * | 0 | 4/6 |
| Cornell | 2020 | * | * | * | * | * | * | 6/6 |
| Pegourie | 2019 | * | 0 | * | * | * | * | 5/6 |
| Storrar | 2019 | * | 0 | 0 | 0 | * | * | 3/6 |
| Li | 2023 | * | * | 0 | * | 0 | 0 | 3/6 |

Risk of bias was evaluated using a modified version of the Newcastle Ottawa Scale. A study can be awarded a maximum of one star for each item, resulting in a final score ranging from 0 to 6. A higher score indicates a higher level of study quality.
^1^ Retrospective cohort
^2^ Prospective cohort

1. **GRADE evidence assessment**

**Table S2.** Assessment of the certainty of evidence.

| **Outcome** | **Number of participants (n cohorts)** | **Certainty of evidence (GRADE)** | **Reason for downgrade** |
| --- | --- | --- | --- |
| *Venous thromboembolism* | 416 (8 cohorts) | ⊕⊝⊝⊝ (very low) | Downgraded to *very low* because of study limitations (no population description, inconsistent follow-up reporting), imprecision (small sample size) and inconsistent results (high heterogeneity). |
| *Minor bleeding* | 192 (5 cohorts) | ⊕⊝⊝⊝ (very low) | Downgraded to *very low* because of imprecision (small sample size), study limitations (lack of follow-up and no population description), inconsistent results (moderate heterogeneity). |
| *Clinically relevant non-major bleeding* | 334 (7 cohorts) | ⊕⊝⊝⊝ (very low) | Downgraded to *very low* because of imprecision (small sample size) and study limitations (lack of follow-up and no population description). |
| *Major bleeding* | 416 (8 cohorts) | ⊕⊝⊝⊝ (very low) | Downgraded to *very low* because of imprecision (small sample size) and study limitations (lack of follow-up and no patient population description). |
| *Arterial thrombosis* | 241 (4 cohorts) | ⊕⊝⊝⊝ (very low) | Downgraded to *very low* because of imprecision (small sample size) and study limitations (lack of follow-up and no patient population description). |
| *Mortality* | 120 (2 cohorts) | ⊕⊝⊝⊝ (very low) | Downgraded to *very low* because of imprecision (very small sample size) and study limitations (lack of follow-up and no population description). |
